# Comparison of the Effects of Dexamethasone and Magnesium Sulfate Used as Adjuvants on Infraclavicular Brachial Plexus Nerve Block: A Prospective, Double-Blinded Randomized Controlled Trial

**DOI:** 10.7759/cureus.78794

**Published:** 2025-02-09

**Authors:** Münevver Güneş, Haci Yusuf Güneş

**Affiliations:** 1 Department of Anesthesiology and Reanimation, Faculty of Medicine, Van Yuzuncu Yil University, Van, TUR

**Keywords:** 0.5% bupivacaine, adjuvant drugs, dexamethasone sodium phosphate, infraclavicular brachial plexus nerve block, magnesium sulfate

## Abstract

Background

This prospective clinical study aims to compare the effectiveness of lower-dose dexamethasone and magnesium sulfate as adjuvants to bupivacaine in ultrasound-guided infraclavicular brachial plexus block for distal upper limb surgery.

Materials and methods

Ninety patients, aged 18 to 65 years, with American Society of Anesthesiologists (ASA) physical status scores of I or II who underwent distal upper limb surgeries, including the arm, elbow, forearm, and hand surgery under infraclavicular brachial plexus block, were included in the study. The block was performed under ultrasound guidance. Patients were allocated to three groups: Group B received 20 mL of 0.5% bupivacaine combined with 5 mL of 0.9% NaCl; Group B+D received 20 mL of 0.5% bupivacaine combined with 4 mg of dexamethasone and 4 mL of 0.9% NaCl; and Group B+M received 20 mL of 0.5% bupivacaine combined with 150 mg of magnesium sulfate and 4 mL of 0.9% NaCl. Outcomes assessed included sensory and motor block onset times, durations of sensory and motor blocks, duration of analgesia, perioperative hemodynamic parameters, and opioid and non-steroidal anti-inflammatory drug (NSAID) consumption.

Results

Data from 90 patients were analyzed. Demographic characteristics and ASA scores were comparable across groups (p > 0.05). Group B+D demonstrated significantly longer durations of motor block, sensory block, and analgesia compared to Group B and Group B+M (p = 0.001). Moreover, Group B+D showed significantly shorter motor (p = 0.001) and sensory (p = 0.003) block onset times and reduced postoperative opioid and NSAID analgesic consumption, as well as lower VAS scores at 12 and 24 hours postoperatively (p = 0.001).

Conclusion

When added as an adjuvant to bupivacaine, dexamethasone resulted in longer durations of motor and sensory block, shorter onset times for both motor and sensory block, and better postoperative analgesia compared to magnesium sulfate. It also reduced opioid and NSAID consumption during the first 24 hours.

## Introduction

With the increase in outpatient surgeries, regional anesthesia has become a more frequently preferred method. Peripheral nerve blocks can be used not only to provide anesthesia but also to offer postoperative analgesia in patients undergoing general anesthesia. The benefits of regional anesthesia over general anesthesia include maintaining patient consciousness, preserving airway reflexes, reducing the consumption of anesthetic and analgesic drugs, ensuring hemodynamic stability, sympathetic blockade, a lower incidence of postoperative side effects such as nausea and vomiting, and a faster recovery time [1,2]. The widespread use of ultrasonography has led to an increased success rate and a reduction in complication rates for peripheral nerve blocks [3]. Infraclavicular nerve block provides adequate anesthesia and effective postoperative analgesia for distal upper limb surgeries, including the arm, elbow, forearm, and hand surgery, while also allowing for the use of a tourniquet. Compared to the supraclavicular nerve block, the risk of pneumothorax is lower.

Various adjuvants are used in peripheral nerve blocks to extend the duration of postoperative analgesia, shorten the onset time of anesthetic effects, reduce the required local anesthetic dose, and lower the risk of local anesthetic toxicity. In recent years, strategies for multimodal approaches to effective pain management have been developed. While the efficacy of local anesthetics can be enhanced by increasing their dose, this also raises the risk of toxicity. By adding an adjuvant drug, the duration of analgesia and the block can be prolonged without increasing the dose of the local anesthetic, and opioid consumption can be reduced [3,4]. Opioid drugs, sodium bicarbonate, clonidine, epinephrine, verapamil, midazolam, dexmedetomidine, and ketamine are among the commonly used adjuvants today. However, the ideal adjuvant drug remains unidentified. Dexamethasone has been increasingly used as an adjuvant in recent years due to its anti-inflammatory and analgesic effects. It has been reported to prolong the duration of analgesia and reduce postoperative opioid consumption when administered both intravenously and perineurally [5].

Magnesium sulfate (MgSO₄) is an N-methyl-D-aspartate receptor antagonist that exhibits antinociceptive effects by regulating calcium influx into cells. Additionally, magnesium possesses anesthetic protective properties, enhances the efficacy of analgesics, and has antihypertensive effects [6,7]. The effects of high-volume local anesthetics combined with high-dose dexamethasone and magnesium sulfate as adjuvants on peripheral nerve blocks have been extensively investigated in numerous studies. Does the efficacy of the block change when both the volume of the local anesthetic and the doses of dexamethasone and magnesium sulfate are reduced? This study aims to evaluate the effectiveness of lower-volume bupivacaine combined with lower-dose dexamethasone and magnesium sulfate in ultrasound-guided infraclavicular brachial plexus block, focusing on block onset time, block duration, analgesia duration, and analgesic consumption.

## Materials and methods

Ethical considerations

This study, which included ninety patients aged 18 to 65 years who underwent distal upper limb surgery (i.e., arm, elbow, forearm, or hand procedures) performed under an infraclavicular brachial plexus block and whose American Society of Anesthesiologists (ASA) physical status scores were I or II, was approved by the Invasive Clinical Research Ethics Committee of Van Yuzuncu Yil University Faculty of Medicine (registration date and approval number: March 2, 2022, 2022/03-03). This study was registered in Clinical Trials with the number NCT06085417. Written informed consent was obtained from all participants.

Patient inclusion and exclusion criteria

The inclusion criteria were as follows: participants aged 18 to 65 years, classified as American Society of Anesthesiologists (ASA) Physical Status (PS) I or II, scheduled for elective arm, elbow, forearm, or hand surgery, and who provided written and verbal informed consent. The exclusion criteria were as follows: patients aged <18 or >65 years, those who declined regional anesthesia, individuals with ASA classification of III or higher, liver or kidney dysfunction, diabetes mellitus, drug allergies, pregnancy, emergency cases, neuropathic disorders, pneumothorax, a BMI greater than 30, local infection, coagulopathy, cardiopulmonary disease, or a prior history of brachial plexus injury.

Randomization

Ninety patients were randomized into three groups of 30 each using the sealed-envelope method. For all three groups, an equal volume of medication was prepared. The anesthetists who prepared the medication, administered it, and evaluated the patient were different individuals. Group Bupivacaine (Group B) consisted of 30 participants who received 20 mL of 0.5% bupivacaine + 5 mL of 0.9% NaCl. Group Bupivacaine + Dexamethasone (Group B+D) included 30 participants who received 20 mL of 0.5% bupivacaine + 4 mg dexamethasone + 4 cc of 0.9% NaCl. Group Bupivacaine + Magnesium Sulfate (Group B+M) consisted of 30 participants who received 20 mL of 0.5% bupivacaine + 150 mg magnesium sulfate (1 mL of 15% magnesium sulfate, Onfarma, Turkey) + 4 mL of 0.9% NaCl. 

Anesthesia

The patients included in the study were informed about the study and were introduced to the Visual Analogue Scale (VAS) preoperatively. Prior to the administration of an infraclavicular block, vascular access was established using a 20-gauge cannula, and a 0.9% NaCl crystalloid infusion was initiated at a rate of 8 mL/kg/hour. Standard ASA monitoring (electrocardiography (ECG), peripheral oxygen saturation (SpO₂), heart rate (HR), and non-invasive blood pressure monitoring) was conducted for all patients. Patients were positioned in the supine position. The upper extremity planned for surgery was positioned with the arm adducted and the forearm flexed at 90 degrees, with the hand placed on the patient's abdomen. Asepsis was maintained in the area where the block would be applied using 10% povidone-iodine, followed by the covering of the area with a sterile drape. An ultrasound (US) probe (Esaote Europe B.V. Netherlands) with a linear frequency range of 5-12 MHz was covered with a sterile transparent sheath. The US probe was positioned in the sagittal plane, 1 cm below the intersection point between the coracoid process and the clavicle. After visualizing the lateral, posterior, and medial cords surrounding the axillary artery, the peripheral nerve block needle (Stimuplex Ultra, 20 Ga. x 4 inches (100 mm), B. Braun Melsungen AG) was guided toward the 8 o'clock position relative to the axillary artery using an in-plane technique aligned with the US probe. The peripheral nerve stimulator (Stimuplex HNS12, B. Braun Medical Inc., Germany) was set at 1 mA current, 2 MHz frequency, and 0.1 ms stimulation duration. The goal was to observe distal motor responses corresponding to posterior and medial cords. Acceptable distal motor responses were evaluated for the ulnar nerve (flexion of the 5th digit), median nerve (flexion of the 3rd digit), and radial nerve (finger and wrist extension). Once the expected distal motor response was achieved, the peripheral nerve stimulator current was decreased to 0.3 mA to avoid intraneural injection. If the motor response was lost, the local anesthetic was slowly injected, aspirating every 5 mL to exclude intravascular injection. Simultaneously, the US image showed that the local anesthetic surrounded the axillary artery at the 8 o'clock position in a C-shaped manner. The following anesthetic mixtures were administered to the groups: Group B received 20 mL of 0.5% bupivacaine plus 5 mL of 0.9% NaCl (control group); Group B+D received 20 mL of 0.5% bupivacaine plus 4 mg of dexamethasone plus 4 mL of 0.9% NaCl; and Group B+M received 20 mL of 0.5% bupivacaine plus 150 mg of magnesium sulfate plus 4 mL of 0.9% NaCl (study groups).

Outcome measures

The primary outcome of the study was the duration of sensory block. The onset times of motor and sensory blockade, the duration of motor blockade, VAS scores, total opioid and NSAID consumption within the first 24 hours, hemodynamic parameters, and the duration of analgesia were evaluated as secondary outcomes.

The duration of sensory block was measured as the time interval from the onset of complete sensory block to its complete resolution.

The onset time of sensory block was defined as the time interval from the injection of the study drug to the achievement of complete sensory blockade.

The duration of analgesia was defined as the time interval from the onset of complete sensory block to the time when the VAS score reached ≥4.

The onset time of motor block was defined as the time interval from the injection of the study medication to the achievement of complete motor blockade.

The duration of motor block was measured as the time interval from the onset of complete motor block to its complete resolution.

After the block was applied, sensory and motor block assessments were performed every five minutes for 30 minutes until complete block was achieved. Sensory block onset was evaluated using the pinprick test, applied to the first three fingers and dorsal wrist (radial nerve), medial side of the fifth finger (ulnar nerve), first three fingers and volar wrist (median nerve), and the lateral forearm (musculocutaneous nerve). The pinprick test was scored as follows: normal sensation, no block = 0; loss of sensation to pinprick or decreased sensation, partial block = 1; and loss of touch sensation or complete loss of sensation, complete block= 2 [8]. Motor function was assessed using a modified Bromage scale, where a score of 3 indicated elbow extension against gravity, 2 indicated wrist flexion against gravity, 1 indicated finger movement, and 0 indicated no movement [9]. A successful block was defined as a modified Bromage scale score of ≤1 and a pinprick sensory test score of ≥1. At the end of 30 minutes, patients with insufficient sensory block were administered 1 mcg/kg fentanyl intravenously for sedation and analgesia. Despite the additional fentanyl, patients experiencing pain were considered to have an unsuccessful block. In the event of an unsuccessful block, general anesthesia was administered, and these patients were excluded from the study.

The VAS score was used to assess pain levels at 0, 3, 6, 12, and 24 hours postoperatively. Patients with a postoperative VAS score of 4 or higher received 50 mg of dexketoprofen trometamol intravenously as rescue analgesia. If the VAS score remained 6 or higher despite dexketoprofen trometamol administration, intravenous tramadol hydrochloride (1 mg/kg) was administered as an opioid analgesic. The patients' sensory and motor block onset times were recorded. In the postoperative period, sensory and motor block resolution times, VAS scores at the 0th, 3rd, 6th, 12th, and 24th hours, total opioid and NSAID consumption in the first 24 hours, and duration of analgesia were recorded. Patients' hemodynamic parameters (heart rate, systolic, diastolic, and mean blood pressures) and SpO₂ values were recorded at baseline (pre-block) and at 5, 15, 30, and 60 minutes post block. All patients were assessed for potential complications such as hematoma, nerve damage, and pneumothorax at 24 hours postoperatively in the ward and one week after discharge in the outpatient clinic.

Statistical analysis

The duration of sensory blocks (in hours) was considered the primary outcome variable in this study. According to previous research [9], the standard deviation (σ) for the duration of sensory blocks ranged from 1.05 to 1.2. Therefore, the standard deviation was assumed to be 1.12 hours for sensory block duration. The sample size was determined based on an 80% power (α = 0.05 and β = 0.20), an effect size (d) of 0.4, and a Z-value of 1.96. Using the formula n = Z²σ² / d², the minimum required sample size was calculated to be 30. Descriptive statistics for continuous variables were presented as the mean, standard deviation, minimum, and maximum values, while categorical variables were summarized using counts and percentages. The normality assumption for continuous variables was tested using the Kolmogorov-Smirnov test. Based on the normality test results, a one-way ANOVA was performed for normally distributed variables, whereas the Kruskal-Wallis test was applied for non-normally distributed variables. Tukey's multiple comparison test was used to identify significant group differences. A significance level of 5% was considered, and all statistical analyses were conducted using IBM SPSS Statistics for Windows, Version 28 (Released 2021; IBM Corp., Armonk, New York, United States).

## Results

**Figure 1:**
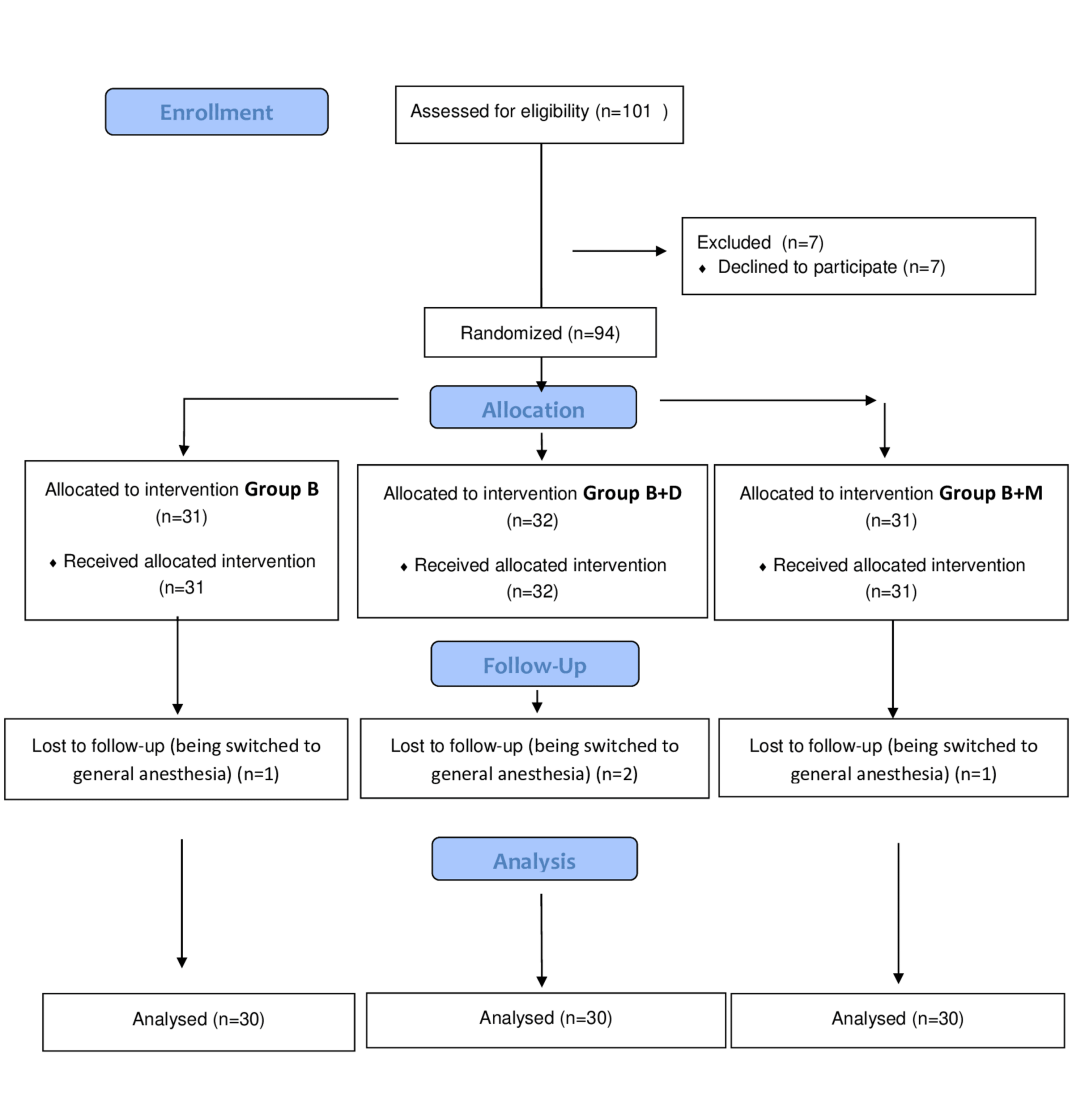
CONSORT Flowchart CONSORT: Consolidated Standards of Reporting Trials

In this study, 101 participants who met the inclusion criteria were evaluated. However, seven participants were excluded because they refused to participate, and four were excluded after being switched to general anesthesia. Data from 90 patients, who were randomized into three groups, were included in the analysis. The demographic characteristics of the patients studied, including age, height, body weight, and BMI distribution rates, were comparable across the three groups. ASA scores were also similar between the groups (Figure 1; Table 1). 

**Table 1:** Demographic Characteristics of the Patients Studied BMI: Body mass index, ASA: American Society of Anesthesiologists, X2: Chi-square, k: Kruskal-Wallis (Mann-Whitney U test), a: ANOVA, SD: Standard deviation. Data is presented as mean ± SD, median or frequency (%).

Variables		Group B n = 30	Group B+D n = 30	Group B+M n = 30	p
Age (year)	Mean ± SD, Median	33.2 ± 14.2, 28.5	33.7 ± 12.0, 31.5	33.4 ± 13.0, 30.0	0.887^k^
Height (cm)	Mean ± SD, Median	172.2 ± 7.9, 171.0	170.7 ± 8.8, 173.0	172.2 ± 6.7, 171.5	0.801^k^
Weight (kg)	Mean ± SD, Median	70.9 ± 13.3, 70.0	70.5 ± 11.0, 72.0	73.4 ± 10.6, 74.0	0.598^a^
BMI	Mean ± SD, Median	23.8 ± 3.4, 23.8	24.1 ± 2.8, 24.6	24.7 ± 2.6, 24.7	0.414^k^
ASA Score	I n-%	13 43.3%	15 50.0%	18 60.0%	0.430^ X²^
	II n-%	17 56.7%	15 50.0%	12 40.0%	

Compared to Group B+D and Group B+M, the motor block onset time (p = 0.001) and sensory block onset time (p = 0.003) were statistically significantly longer in Group B. However, no statistically significant differences were observed between Group B+D and Group B+M in terms of motor block and sensory block onset times (Table 2).

**Table 2:** Onset Time and Duration of Motor and Sensory Block in the Groups SD: Standard deviation, k: Kruskal-Wallis (Mann-Whitney U test). Data is presented as mean ± SD, Median

Variables	Group B (n 30) Mean ± SD, Median	Group B+D (n 30) Mean ± SD, Median	Group B+M (n 30) Mean ± SD, Median	p
Onset time of block (minute)				
Motor block onset time	14.6 ± 3.7, 14.5	10.6 ± 1.9, 10.0	12.3 ± 3.2, 12.0	0.000^k^
Sensory block onset	11.0 ± 4.2, 10.0	7.6 ± 2.2, 7.0	9.0 ± 3.2, 9.0	0.003^k^
Duration of block (hour)				
Duration of motor block	13.7 ± 4.8, 12.5	22.4 ± 7.6, 22.0	16.8 ± 5.6, 16.0	0.000^k^
Duration of sensory block	14.5 ± 4.9, 13.0	23.3 ± 8.0, 23.0	17.7 ± 6.2, 16.5	0.000^k^

When comparing motor block durations across groups, the longest motor block duration was observed in Group B+D (22.4 ± 7.6 hours), while the shortest was in Group B (13.7 ± 4.8 hours). In Group B+M, the motor block duration was 16.8 ± 5.6 hours. The motor block duration in Group B+D was statistically significantly higher than that in both Group B and Group B+M (p = 0.001). Additionally, when comparing Group B with Group B+M, motor block durations were found to be statistically significantly higher in Group B+M (p = 0.001). The sensory block duration was statistically significantly longer in Group B+D compared to both Group B and Group B+M (p = 0.001). Additionally, the sensory block duration in Group B+M was statistically significantly higher than that in Group B (p = 0.001) (Table 2).

**Figure 2:**
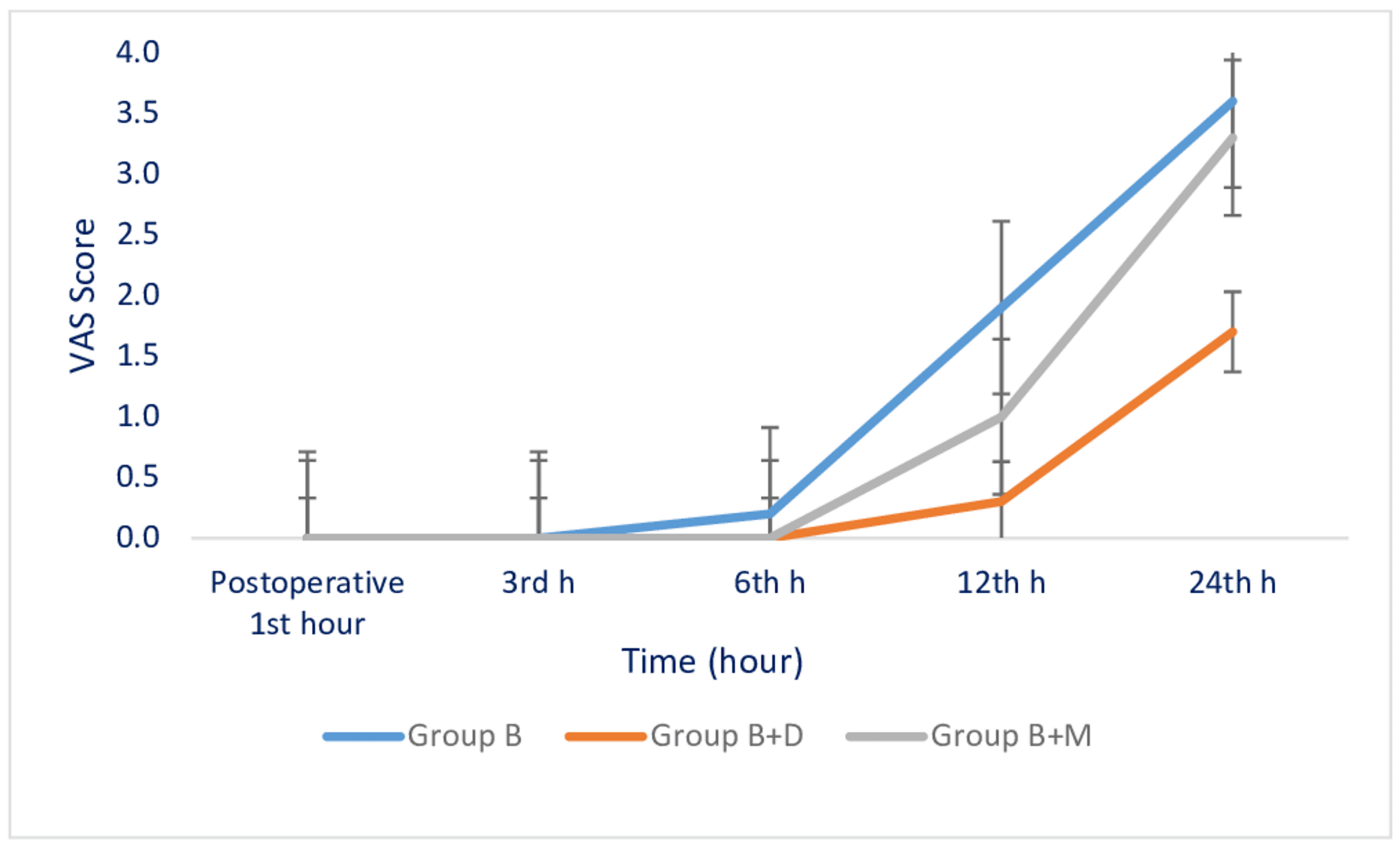
Comparison of Postoperative VAS Scores Between Groups VAS: Visual analogue scale

VAS scores at postoperative first, third, and sixth hours were similar across all three groups. In Group B, the VAS score at the postoperative 12th hour was statistically significantly higher compared to both Group B+D and Group B+M (p = 0.001). There were no significant differences between Group B+D and Group B+M in terms of the postoperative 12th-hour VAS score. The postoperative 24th-hour VAS score in Group B was statistically significantly higher than that in the other two groups (Group B+D and Group B+M) (p = 0.001). Additionally, the postoperative 24th-hour VAS score in Group B+M was also statistically significantly higher than that in Group B+D (p = 0.001) (Table 3, Figure 2). 

**Table 3:** Comparison of VAS Scores Among Groups VAS: Visual analogue scale, k: Kruskal-Wallis (Mann-Whitney U test), SD: Standard deviation. Data is presented as mean ± SD, median or frequency (%).

Variables	Group B (n 30) Mean ± SD, Median	Group B+D (n 30) Mean ± SD, Median	Group B+M (n 30) Mean ± SD, Median	p
VAS Score- Postoperative				
1st hour	0.0 ± 0.0, 0.0	0.0 ± 0.0, 0.0	0.0 ± 0.0, 0.0	1.000^k^
3rd hour	0.0 ± 0.0, 0.0	0.0 ± 0.0, 0.0	0.0 ± 0.0, 0.0	1.000^k^
6st hour	0.2 ± 0.5, 0.0	0.0 ± 0.0, 0.0	0.0 ± 0.0, 0.0	0.051^k^
12th hour	1.9 ± 1.1, 2.0	0.3 ± 0.8, 0.0	1.0 ± 1.7, 0.0	0.001^k^
24th hour	3.6 ± 1.8, 4.0	1.7 ± 1.5, 2.0	3.3 ± 1.4, 4.0	0.000^k^

The number of patients requiring postoperative opioids and NSAIDs was significantly higher in Group B compared to Group B+D and Group B+M (p = 0.021). In Group B+M, the number of patients requiring postoperative opioids and NSAIDs was higher than that in Group B+D. However, this difference was not statistically significant. In Group B+D, the analgesia duration was statistically significantly longer compared to both Group B and Group B+M (p = 0.001). When compared to Group B, Group B+M also showed a statistically significantly longer analgesia duration (p = 0.001) (Table 4).

**Table 4:** Comparison of sedation requirement, postoperative analgesic consumption rate, and duration of analgesia among groups NSAID: Non-steroidal anti-inflammatory drug, X2: Chi-square, k: Kruskal-Wallis (Mann-Whitney U test), SD: Standard deviation. Data is presented as mean ± SD, median or frequency (%).

Variables			Group B (n 30)		Group B+D (n 30)		Group B+M (n 30)		p
Intraoperative additional sedation requirement	(-)	n -%	24	80.0%	29	96.7%	26	86.7%	0.140^ X²^
	(+)	n -%	6	20.0%	1	3.3%	4	13.3%	
Postoperative opioid requirement	(-)	n -%	21	70.0%	29	96.7%	25	83.3%	0.021^ X²^
	(+)	n -%	9	30.0%	1	3.3%	5	16.7%	
Postoperative NSAID requirement	(-)	n -%	4	13.3%	18	60.0%	8	26.7%	0.000^ X²^
	(+)	n -%	26	86.7%	12	40.0%	22	73.3%	
Duration of analgesia (hours)		Mean ± SD	12.7 ± 4.7		21.8 ± 7.5		15.8 ± 6.1		0.000^ X²^
		Median	12.0		21.0		14.5		

**Figure 3:**
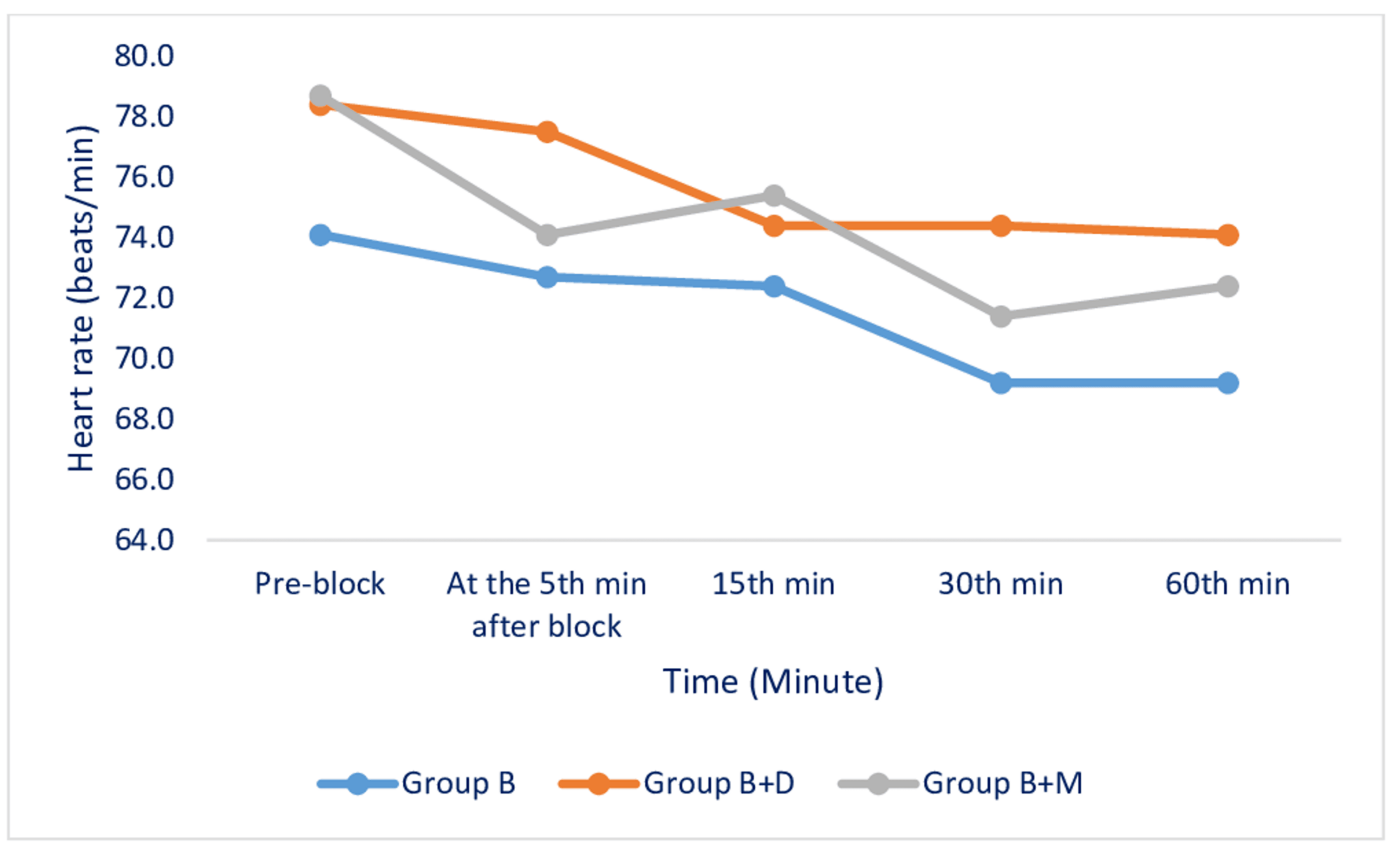
Changes in the Heart Rate Among the Studied Groups VAS: Visual analogue scale

**Figure 4:**
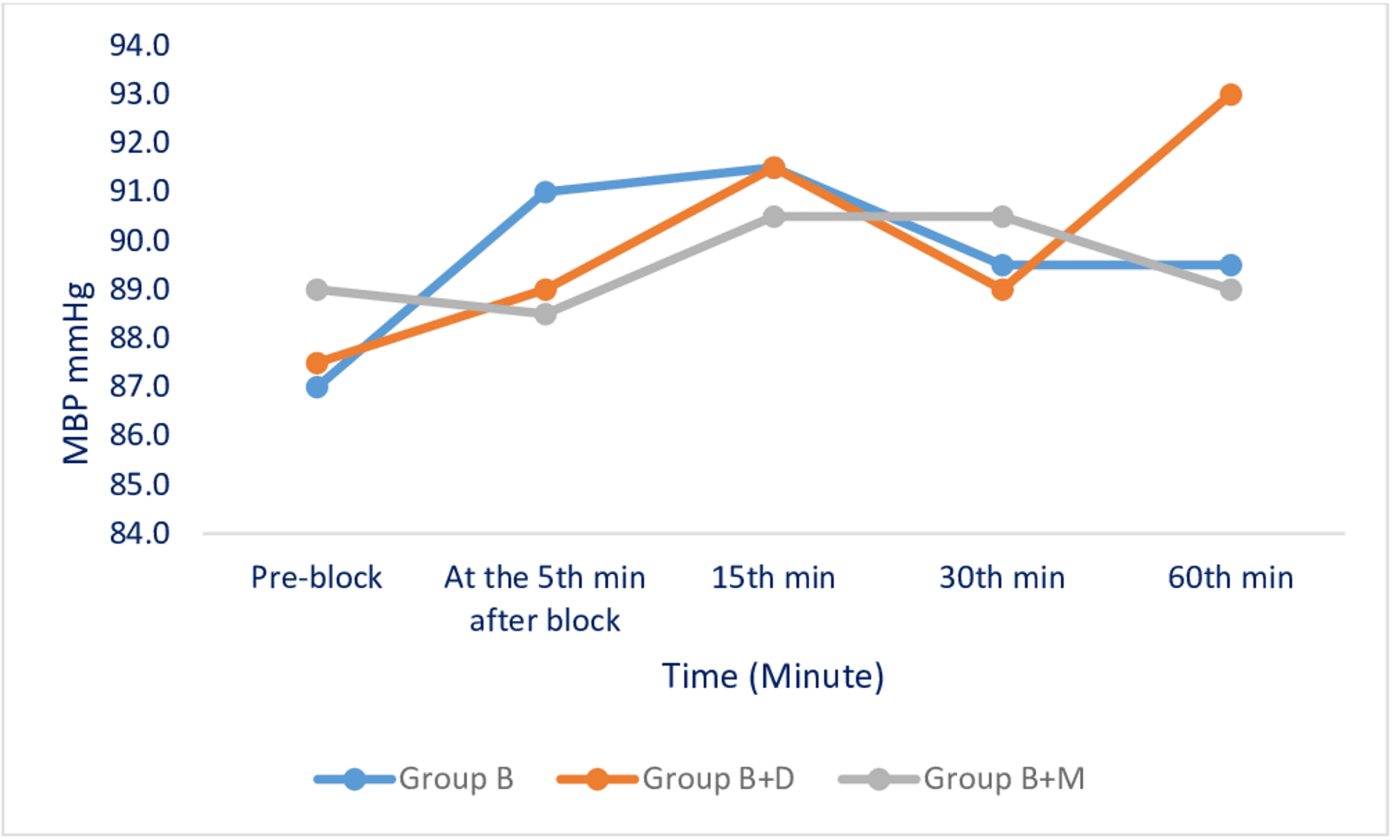
Changes in Mean Blood Pressure (MBP) Among the Studied Groups

HR (Figure 3), mean blood pressure (Figure 4), and SpO₂ values measured during the perioperative period were similar among the groups. No side effects were observed during the perioperative period.

## Discussion

The main finding of this study is that the group in which dexamethasone was added as an adjuvant to bupivacaine exhibited longer durations of motor block, sensory block, and analgesia compared to the control group and the group with added magnesium sulfate. Moreover, motor and sensory block onset times, postoperative consumption of opioids and NSAID analgesics, and VAS scores at the 24th hour were all lower in this group.

In regional anesthesia, adjuvant agents have been used since 1982 to prolong block duration and enhance the quality of anesthesia and analgesia. While used alone in regional blocks, they do not provide significant analgesic effects. However, when added as adjuvants to local anesthetics, such as dexamethasone and magnesium sulfate, they extend the duration of peripheral nerve blocks and enhance the quality of the block [10]. Steroids are believed to prolong both the duration of the block and the analgesic effect through mechanisms such as reducing the systemic absorption of local anesthetics, suppressing C-fiber transmission of pain signals, and attenuating the inflammatory response [11,12]. Magnesium sulfate, an NMDA antagonist, is thought to play a role in regulating calcium influx into neurons, influencing neurotransmitter release at the synaptic gap, thereby potentiating the effects of local anesthetics [13]. Magnesium sulfate, when added as an adjuvant to bupivacaine hydrochloride, has been reported to enhance the efficacy of the transversus abdominis plane block and prolong the duration of analgesia. In superficial cervical plexus blocks, the adjuvant agent dexamethasone added to bupivacaine has been reported to prolong postoperative analgesia duration [14,15]. Intrathecal administration of dexamethasone has been reported to prolong the duration of sensory block and postoperative analgesia without increasing the frequency of adverse events [16]. Our study compared frequently used adjuvant drugs, dexamethasone and magnesium sulfate. The onset times of motor and sensory blocks were found to be statistically significantly shorter in the dexamethasone group. The duration of sensory block in Group B+D was 8.8 hours longer than in the control group and 5.6 hours longer than in Group B+M, respectively. It was observed that the durations of motor and sensory blocks in the dexamethasone group were statistically significantly longer compared to the magnesium group.

Suboptimal postoperative pain management can contribute to extended hospital stays, higher morbidity rates, and the development of chronic pain, negatively impacting patients' quality of life [17]. Hadzic et al. found that in upper extremity surgeries, infraclavicular brachial plexus block, compared to general anesthesia, reduces the need for additional postoperative analgesia, provides better analgesic scores, results in fewer side effects, and enables earlier ambulation [18]. This has increased anesthesiologists' interest in peripheral nerve blocks for postoperative analgesia, encouraging further research to enhance the analgesic efficacy and duration of peripheral nerve blocks [19]. In a study comparing dexamethasone and magnesium sulfate added as adjuvants to ropivacaine for caudal block, a statistically significant enhancement in the duration of analgesia was reported [20]. In our study, the duration of analgesia was found to be an average of 12.7 hours in the control group, 15.8 hours in the magnesium group, and 21.8 hours in the dexamethasone group. Based on these findings, dexamethasone effectively prolonged the postoperative duration of analgesia and reduced postoperative analgesic requirements more effectively than magnesium.

De Oliveira reported that the addition of dexamethasone as an adjuvant to ropivacaine or bupivacaine reduced postoperative opioid consumption; however, no significant difference was observed between dexamethasone and placebo at postoperative 24th hour [21]. Pehora et al., in their Cochrane review, reported no significant difference between dexamethasone and placebo at postoperative 48th hours. However, they noted that in cases where perineural dexamethasone was administered, both pain and 24-hour opioid consumption were significantly reduced at postoperative 12th and 24th hours [22]. In this study, dexamethasone provided effective analgesia during the first 24 hours and reduced opioid and NSAID analgesic consumption. The low VAS scores at the 12th and 24th hours further support this finding. VAS scores during the first six hours were similar across all three groups. At the 24th hour, the VAS score in the dexamethasone group was significantly lower than in both the magnesium and control groups. Although magnesium sulfate was not as effective as dexamethasone in providing analgesia up to the 24th hour.

Schnepper et al. in their review, reported that very low doses of dexamethasone (2 mg or less) were not as effective as low doses (2-4 mg) [23]. Similarly, Tandoc et al., in their study comparing 4 mg and 8 mg doses of dexamethasone added as an adjuvant to bupivacaine, reported that both doses were equally effective in terms of duration of motor block and analgesia [24]. Gündüz et al. reported that perineural magnesium sulfate prolonged sensory block duration more effectively than intravenous magnesium sulfate. Additionally, 150 mg perineural magnesium sulfate was found to be more effective in prolonging sensory block duration compared to 100 mg perineural magnesium sulfate [25]. In our study, we preferred the low doses of adjuvant agents that were found to be effective in previous research. Our findings were consistent with the results of studies conducted with higher doses [14,15]. These doses effectively prolonged sensory block and analgesia duration while reducing postoperative analgesic consumption. Furthermore, no side effects were observed with these doses.

In this study, the minimum required sample size was set at 30 patients per group. Consequently, 30 patients were assigned to each group. However, the limited patient population was identified as a limitation of the study.

## Conclusions

In conclusion, dexamethasone added as an adjuvant to bupivacaine provided more effective analgesia than magnesium sulfate during the postoperative 24 hours and it reduced opioid and NSAID analgesic consumption. We believe that dexamethasone is a preferable option for increasing the quality and duration of anesthesia and analgesia in peripheral nerve blocks.

## References

[1] Mirza F, Brown AR (2011). Ultrasound-guided regional anesthesia for procedures of the upper extremity. Anesthesiol Res Pract.

[2] O'Donnell BD, Ryan H, O'Sullivan O, Iohom G (2009). Ultrasound-guided axillary brachial plexus block with 20 milliliters local anesthetic mixture versus general anesthesia for upper limb trauma surgery: an observer-blinded, prospective, randomized, controlled trial. Anesth Analg.

[3] Klaastad O, Sauter AR, Dodgson MS (2009). Brachial plexus block with or without ultrasound guidance. Curr Opin Anaesthesiol.

[4] Fernández Martin MT, Alvarez Lopez S, Aldecoa Alvarez-Santullano C (2023). Role of adjuvants in regional anesthesia: a systematic review. Rev Esp Anestesiol Reanim (Engl Ed).

[5] Schäfer M, Mousa SA, Shaqura M, Tafelski S (2019). Background and current use of adjuvants for regional anesthesia: from research to evidence-based patient treatment (Article in German). Anaesthesist.

[6] Soleimanpour H, Imani F, Dolati S, Soleimanpour M, Shahsavarinia K (2022). Management of pain using magnesium sulphate: a narrative review. Postgrad Med.

[7] Li M, Jin S, Zhao X, Xu Z, Ni X, Zhang L, Liu Z (2016). Does magnesium sulfate as an adjuvant of local anesthetics facilitate better effect of perineural nerve blocks?: a meta-analysis of randomized controlled trials. Clin J Pain.

[8] Turner JD, Henshaw DS, Weller RS (2018). Perineural dexamethasone successfully prolongs adductor canal block when assessed by objective pinprick sensory testing: a prospective, randomized, dose-dependent, placebo-controlled equivalency trial. J Clin Anesth.

[9] Ghazaly HF, Aly AA, Zaher ZZ, Hassan MM, Mahmoud AA (2022). Comparison of the efficacy of two doses of dexmedetomidine as an adjunct to levobupivacaine in infraclavicular brachial plexus block: prospective double-blinded randomized controlled trial. BMC Anesthesiol.

[10] Candido KD, Knezevic NN (2011). All adjuvants to local anesthetics were not created equal: animal data evaluating neurotoxicity, thermal hyperalgesia, and relevance to human application. Reg Anesth Pain Med.

[11] Krishna Prasad GV, Khanna S, Jaishree SV (2020). Review of adjuvants to local anesthetics in peripheral nerve blocks: current and future trends. Saudi J Anaesth.

[12] Shishido H, Kikuchi S, Heckman H, Myers RR (2002). Dexamethasone decreases blood flow in normal nerves and dorsal root ganglia. Spine (Phila Pa 1976).

[13] James MF (1992). Clinical use of magnesium infusions in anesthesia. Anesth Analg.

[14] Satish Kumar MN, Archana M, Dayananda VP, Surekha C, Ramachandraiah R (2022). A study to evaluate the efficacy of dexamethasone as an adjuvant in ultrasound-guided bilateral superficial cervical plexus block using 0.25% bupivacaine in patients undergoing thyroid surgeries under entropy-guided general anesthesia. Anesth Essays Res.

[15] Ammar AS, Mahmoud KM, Kasemy ZA (2018). Comparison between adenosine and magnesium sulphate as adjuvants for transversus abdominis plane block: a prospective randomized controlled trial. Minerva Anestesiol.

[16] Pascarella G, Ruggiero A, Garo ML (2024). Intrathecal dexamethasone as an adjuvant for spinal anesthesia: a systematic review. Minerva Anestesiol.

[17] Gan TJ (2017). Poorly controlled postoperative pain: prevalence, consequences, and prevention. J Pain Res.

[18] Hadzic A, Arliss J, Kerimoglu B (2004). A comparison of infraclavicular nerve block versus general anesthesia for hand and wrist day-case surgeries. Anesthesiology.

[19] Aveline C, Le Hetet H, Le Roux A (2011). Perineural ultrasound-guided catheter bacterial colonization: a prospective evaluation in 747 cases. Reg Anesth Pain Med.

[20] Yousef GT, Ibrahim TH, Khder A, Ibrahim M (2014). Enhancement of ropivacaine caudal analgesia using dexamethasone or magnesium in children undergoing inguinal hernia repair. Anesth Essays Res.

[21] De Oliveira GS Jr, Castro Alves LJ, Nader A, Kendall MC, Rahangdale R, McCarthy RJ (2014). Perineural dexamethasone to improve postoperative analgesia with peripheral nerve blocks: a meta-analysis of randomized controlled trials. Pain Res Treat.

[22] Pehora C, Pearson AM, Kaushal A, Crawford MW, Johnston B (2017). Dexamethasone as an adjuvant to peripheral nerve block. Cochrane Database Syst Rev.

[23] Schnepper GD, Kightlinger BI, Jiang Y, Wolf BJ, Bolin ED, Wilson SH (2018). A retrospective study evaluating the effect of low doses of perineural dexamethasone on ropivacaine brachial plexus peripheral nerve block analgesic duration. Pain Med.

[24] Tandoc MN, Fan L, Kolesnikov S, Kruglov A, Nader ND (2011). Adjuvant dexamethasone with bupivacaine prolongs the duration of interscalene block: a prospective randomized trial. J Anesth.

[25] Gunduz A, Bilir A, Gulec S (2006). Magnesium added to prilocaine prolongs the duration of axillary plexus block. Reg Anesth Pain Med.

